# A flexible approach to measure care coordination based on patient-sharing networks

**DOI:** 10.1186/s12874-023-02106-0

**Published:** 2024-01-03

**Authors:** Alexander Engels, Claudia Konnopka, Espen Henken, Martin Härter, Hans-Helmut König

**Affiliations:** 1 Department of Health Economics and Health Services Research, Center for Psychosocial Medicine, University Medical Center Hamburg-Eppendorf, Hamburg, Germany; 2 Department of Medical Psychology, Center for Psychosocial Medicine, University Medical Center Hamburg-Eppendorf, Hamburg, Germany

**Keywords:** Care coordination, Provider social networks, Care density, Continuity of care, Claims data analysis

## Abstract

**Background:**

Effective care coordination may increase clinical efficiency, but its measurement remains difficult. The established metric “care density” (CD) measures care coordination based on patient-sharing among physicians, but it may be too rigid to generalize across disorders and countries. Therefore, we propose an extension called fragmented care density (FCD), which allows varying weights for connections between different types of providers. We compare both metrics in their ability to predict hospitalizations due to schizophrenia.

**Methods:**

We conducted a longitudinal cohort study based on German claims data from 2014 through 2017 to predict quarterly hospital admissions. 21,016 patients with schizophrenia from the federal state Baden-Württemberg were included. CD and FCD were calculated based on patient-sharing networks. The weights of FCD were optimized to predict hospital admissions during the first year of a 24-month follow-up. Subsequently, we employed likelihood ratio tests to assess whether adding either CD or FCD improved a baseline model with control variables for the second follow-up year.

**Results:**

The inclusion of FCD significantly improved the baseline model, Χ^2^(1) = 53.30, *p* < 0.001. We found that patients with lower percentiles in FCD had an up to 21% lower hospitalization risk than those with median or higher values, whereas CD did not affect the risk.

**Conclusions:**

FCD is an adaptive metric that can weight provider relationships based on their relevance for predicting any outcome. We used it to better understand which medical specialties need to be involved to reduce hospitalization risk for patients with schizophrenia. As FCD can be modified for different health conditions and systems, it is broadly applicable and might help to identify barriers and promoting factors for effective collaboration.

**Supplementary Information:**

The online version contains supplementary material available at 10.1186/s12874-023-02106-0.

## Introduction

The number of patients affected by chronic diseases continues to rise due to the ongoing demographic shift towards an older population [1, 2]. Therefore, it is important to optimize care coordination as one potential mechanism to improve efficient chronic disease management. Fragmentation and isolated delivery of individual services lead to poor outcomes and inefficient provision of care [3, 4], while the coordination of a patient’s care from disparate providers may improve health outcomes and reduce spending. However, it is unclear how to incentivize effective coordination. In the US, the effects on costs of the first evaluated care coordination programs were disappointing [5]. Moreover, some authors believe that the implementation costs for coordinated care offset potential cost reductions resulting from the prevention of e.g. hospital admissions [6], medical errors or other adverse events [7, 8]. Nevertheless, some care coordination programs such as accountable care organizations or patient-centered medical homes are becoming more and more prevalent in the US [9, 10].

Despite the potential advantages of coordinated care, it remains difficult to robustly measure the resulting effects and to identify the promoting and inhibiting factors that determine the success of care coordination programs. To address this challenge, a metric called care density (CD) was proposed in 2013 to measure outpatient care coordination (OCC) based on readily available claims data [11]. CD assesses patient-sharing among office-based physicians because empirical evidence suggests that shared patients in claims – i.e. billing services to the same patients – are associated with self-reported professional relationships between providers [12]. Therefore, CD assumes that OCC can be approximated by calculating the average number of shared patients across the provider pairs within a patient’s network. The metric was related to quality measures and/or expenditures for a broad range of disorders including cancer [13], congestive heart failure, chronic obstructive pulmonary disease and diabetes [11, 14]. Hence, patient-sharing networks and CD might pose a promising technique to understand and support OCC.

Nevertheless, CD has some critical limitations. CD assumes that all relationships, which share the same amount of patients, contribute equally to care coordination. However, shared patients between providers with a low total patient volume may be more suggestive of a true professional relationship than the same number of shared patients between providers with a high patient volume. Moreover, how much each of the different provider relationships contribute to a patient’s recovery may vary between disorders and outcomes. While the involvement of a psychiatrist may be essential for effectively treating severe depression [15], it may not be necessary for treating mild and moderate cases. Similarly, we would assume diverging effects for the relationship between inpatient and outpatient providers, which may be crucial for preventing readmissions [14], but less so for preventing first time admissions. CD is not versatile enough to account for context-dependent effects. Furthermore, CD depends on the number of shared patients, which are prone to outliers and affected by factors besides coordination.

To overcome these limitations, we propose an extension of CD that can incorporate control variables, outliers, and non-linear effects in the number of shared patients as well as varying effects of different connection types. To explore the added value of this extension, we determined both CD and our extension using German claims data and compared their ability to explain hospitalizations of patients with schizophrenia. We chose patients with schizophrenia as an example, because it is a severe and chronic mental disorder with high hospitalization rates [16–18], albeit efficient OCC may reduce hospitalizations and thus inpatient costs [19].

## Methods

### Data source

Administrative claims data from 2014 through 2017 provided by the Allgemeine Ortskrankenkasse Baden-Württemberg which is a German statutory health insurance responsible for approximately 4.5 million insured individuals.

### Cohort definition

Following Pollack, Weissman, we defined a nested cohort. It consists of a cohort of psychiatric patients and a subcohort that merely includes patients with schizophrenia. This distinction was motivated by the assumption that clinicians tend to cooperate (or not) irrespective of the specific disorder. Therefore, we considered a range of common mental disorders that require collaboration between providers to determine the amount of patient sharing between providers (see Supplemental Table 1 for details). Subsequently, we constructed patient sharing networks based on this information and assessed whether patient-level network metrics were able to predict hospitalizations in the subcohort of patients with schizophrenia. Regardless of the cohort type, we used diagnoses from hospitals, psychiatric or university outpatient clinics and mental health specialists. Other outpatient diagnoses were only considered if they were documented in two successive quarters because the validity of single outpatient diagnoses in claims is limited [20, 21]. All patients lived in the German federal state Baden-Württemberg and had continuous insurance coverage for the study period.

### Provider networks based on patient-sharing

We selected providers who might be involved in the treatment of schizophrenia – i.e. psychiatrists, psychotherapists, neurologists and general practitioners (GPs). Some physicians will be referenced as neurologist/psychiatrist, because the two disciplines used to be unified in Germany until 1994. We excluded providers with an office outside of Baden-Württemberg if they provided fewer services than 95% of the providers inside of Baden-Württemberg with the same specialty, since these were presumably not visited regularly by the patients in our sample.

For each provider pair $i$ we determined the number of shared patients ${w}_{i}$ per calendar year – i.e., patients in the psychiatric cohort who utilized services of both providers – because the likelihood that physicians will adopt a professional relationship increases with ${w}_{i}$ [12]. It is still debated how many shared patients represent an important connection [22, 23]. We decided on a small threshold of ${w}_{i}\ge 3$ to retain connections with psychotherapists who share few patients due to their low patient volume. This absolute threshold was used for the entire network and all provider specialties. Afterwards, we constructed so-called patient-centred networks based on this information for each patient in the subcohort with schizophrenia. These networks consist solely of the providers of a particular patient.

**Fig. 1:**
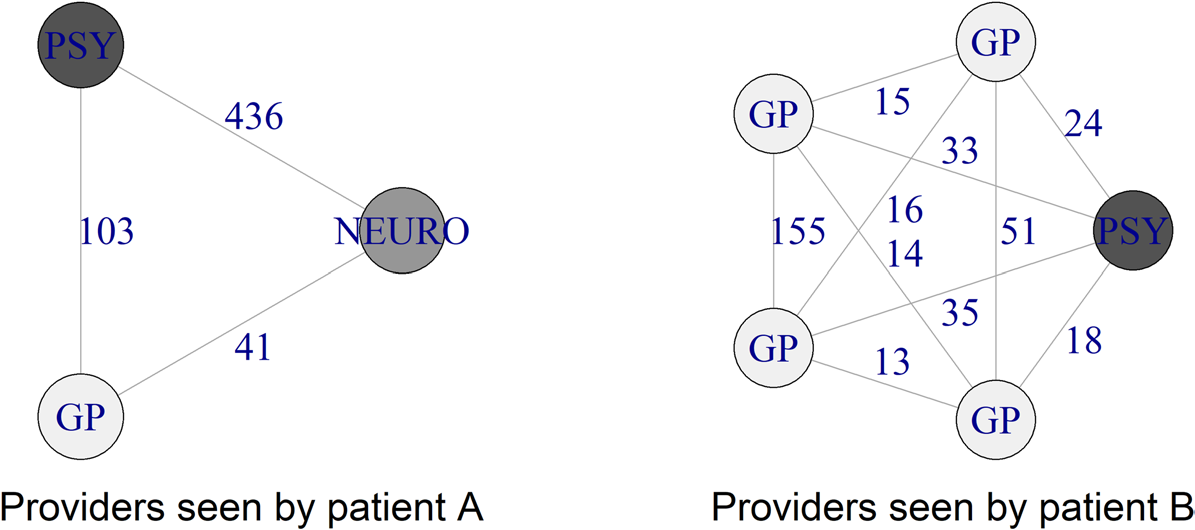
Two empirical examples of patient-centred networks *Notes*: GP General practitioner, PSY psychiatrist, NEURO neurologist, the two examples show the patient-centered networks of two different patients with schizophrenia in one of the quarters in 2015, the nodes represent providers seen by the patient and the numbers adjacent to the links represent the number of psychiatric patients who utilized services from both providers

As an illustration, Fig. 1 displays two of these networks for the year 2015.

The links represent the number of shared patients (${w}_{i}$) between the providers in the corresponding calendar year. We assume that coordination between the providers of patient B is more cumbersome due to the numerous GPs involved, while patient A benefits from an interdisciplinary, compact and strongly connected network. We would argue that it is desirable to have one GP who feels responsible for one patient, knows the individual medical history, especially for chronically ill patients with somatic comorbidities, and acts as a coordinator of care.

### Patient-level network measures

#### Care density

Patient-centred networks have to be aggregated to predict patient-level outcomes. To that end, care density (${C}_{p}$) [11] calculates the average number of shared patients within a patient’s network:

1 $${C}_{p}= \frac{\sum_{i=1}^{m}{w}_{i}}{{n}_{p}({n}_{p}-1)/2}$$

In this formula, ${w}_{i}$ is the number of shared patients of provider pair $i$, n_p_ is the number of providers of patient $p$ and $m$ is the number of provider pairs (Fig. 1 shows that $m=3$ for patient A and $m=10$ for patient B). However, ${C}_{p}$ has several undesirable properties.

One property of ${C}_{p}$ is that the contribution of each provider pair to the total value of ${C}_{p}$ depends solely on how many patients were shared, which causes care density to overstate the relevance of connections between providers with a high patient volume. As a result, in Germany, connections between GPs will strongly increase care density because GPs typically have a high patient volume and thus will on average also share more patients than specialists. However, having more than one GP is a known predictor for potentially avoidable hospitalization [24]. Moreover, favouring networks with multiple GPs contradicts most frequently used continuity of care metrics, because these tend to favour stability with regard to the primary care provider [25]. Another concern is that ${C}_{p}$ cannot incorporate that some connection types may be more important for managing the disorder of interest. Patients with schizophrenia may benefit in particular from connections between GPs and psychiatrists, because these providers need to coordinate to identify and manage somatic comorbidities and guarantee efficient long-term monitoring [26, 27]. Therefore, an adjusted metric should allow for separate effects for each of the $k$ unique connection types and account for the fact that ${w}_{i}$ is correlated with other factors besides patient-related coordination (e.g. regional proximity, patient volume). To allow for separate effects for each of the $k$ unique connection types j, we extend the original approach of ${C}_{p}$.

#### Fragmented care density: an extension of care density

We determined $k$ using the formula $$k=\left(\begin{array}{c}l\\ 2\end{array}\right)+l$$, where $l$ is the number of provider types and which assumes that same specialty connections are of interest. In this study $k=15$, because we can obtain 15 unique provider type combinations by repeatedly selecting from the pool of included provider types (i.e. psychiatrists, neurologist/psychiatrist, neurologist, psychotherapist, and GP). Moreover, we derived an alternative formula for ${C}_{p}$ that begins by calculating the sum of ${w}_{i}$ for each unique connection type j. These sums are labelled ${s}_{j}$ and can easily be calculated in matrix notation if we introduce a dummy ${d}_{ji}$:

2 $$s_j=\begin{pmatrix}w_1\\\vdots\\w_m\end{pmatrix}\cdot\begin{pmatrix}d_{j1}&\dots&d_{jm}\end{pmatrix},$$

where ${d}_{ji}$ indicates whether the provider pair $i$ is of type $\mathrm{j}$:

3 $$d_{ji}=\left\{\begin{array}{l}1\;\mathrm{if}\;\mathrm{provider}\;\mathrm{pair}\;\mathrm i\;\mathrm{is}\;\mathrm{of}\;\mathrm{type}\;\mathrm j\\0\;\mathrm{if}\;\mathrm{provider}\;\mathrm{pair}\;\mathrm i\;\mathrm{is}\;\mathrm{not}\;\mathrm{of}\;\mathrm{type}\;\mathrm j\end{array}\right.$$

Subsequently, ${C}_{p}$ can be written as follows:

4 $${C}_{p}= \frac{\sum_{i=1}^{m}{w}_{i}}{{n}_{p}({n}_{p}-1)/2}= \sum_{i=1}^{m}\frac{{w}_{i}}{{n}_{p}({n}_{p}-1)/2}= \sum_{j=1}^{k}\frac{{s}_{j}}{{n}_{p}({n}_{p}-1)/2}$$

The main benefit of this alternative formula is that each patient’s network can be described using $k$ different sums ${s}_{j}/({n}_{p}({n}_{p}-1)/2)$. Through the conversion of each patient’s network into $k$ variables, irrespective of network size and the number of connections, it becomes easy to introduce connection type specific weights ${\upbeta }_{j}$. These weights ${\upbeta }_{j}$ can be estimated using regression techniques if we define an optimization criterion. For instance, if we optimize the weights to predict a binary outcome, we could apply a logistic regression:

5 $${\mathit{FC}}_{p}=\mathrm{ ln}\left(\frac{p\left(y=1\right)}{1-p\left(y=1\right)}\right)={\beta }_{0}+\sum_{j=1}^{k}{\beta }_{j}\frac{{s}_{j}}{{n}_{p}({n}_{p}-1)/2}$$

Another benefit of the use of regression techniques is that control variables can be included that adjust for factors that might increase network size or the number of shared patients, but are unrelated to patient-related coordination (e.g. the severity of the condition, physician or population density). We labelled the weighted version of ${C}_{p}$ fragmented care density (${FC}_{p}$) as it starts by decomposing ${C}_{p}$ into $k$ variables. In this study, we optimized ${\upbeta }_{j}$ to explain hospital admissions due to schizophrenia.

Furthermore, we decided to categorize each of the sums ${s}_{j}/({n}_{p}({n}_{p}-1)/2$) into tertile-based dummy indicators to allow for non-linear effects of the different connection types and to alleviate the influence of outliers, which are rather common in German claims data (e.g. due to group practices). The indicators encode whether a connection is missing (reference category) or falls into the first (weak link), second (moderate link), or third tertile (strong link) with regard to the connection type specific sum. This approach circumvents the problematic assumption of ${C}_{p}$ that each additional shared patient contributes equally to care coordination (i.e. a linear relationship), although the relationship is presumably non-linear [12]. The only downside is that we must estimate three times as many weights, which might be difficult in smaller samples.

In Supplemental Fig. 1, we summarize the steps that are necessary to convert ${C}_{p}$ to ${FC}_{p}$. We also highlight the main differences between the two metrics using the network of patient B as an example.

#### Study design to compare both metrics

So far, we mainly assessed the psychiatric cohort to determine network ties between physicians, but all subsequent steps will be concerned with the subcohort. We included patients who were diagnosed with schizophrenia in one of the quarters of 2015. The exact study period varied between individuals, because the individual 24- months follow-up period started in the quarter of the diagnoses. The outcome was hospitalizations due to schizophrenia or a common comorbidity – i.e. alcohol or drug abuse, depression, social phobia and posttraumatic stress, generalized anxiety, or obsessive–compulsive disorder [28]. Pre-existing comorbidities were assessed using most Elixhauser subscales [29] based on the 12-months preceding the quarter of the diagnosis. Patients who died during the observation period were excluded in order to avoid bias to the network metrics, because after death the lack of a network would be associated with a decreased risk of hospitalization, although patients were simply no longer able to visit providers or be hospitalized.

**Fig. 2:**
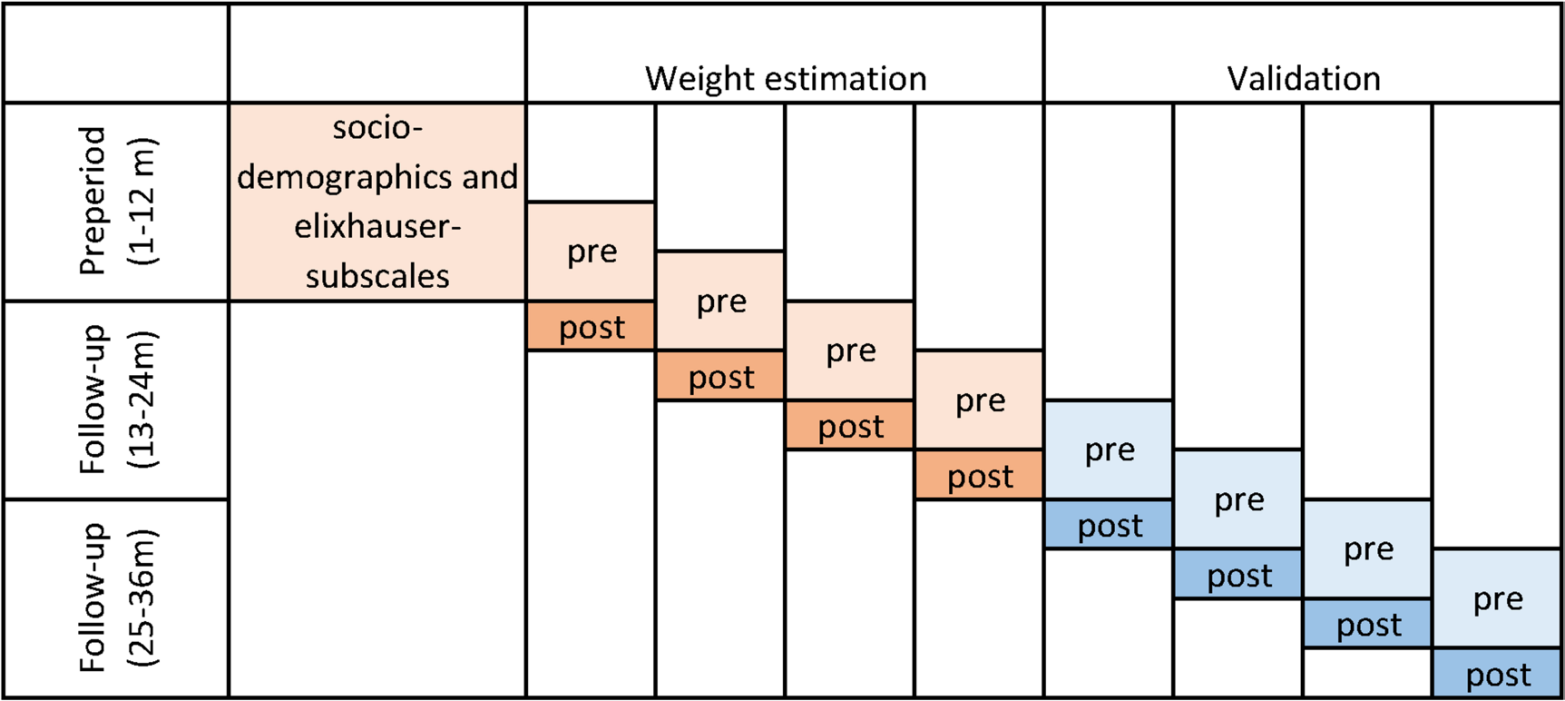
Study design *Notes*: The post / follow-up period was devided into eight quarters, in which we determined hospitalizations due to schizophrenia as our outcome. All time-fixed control variables were dertermined in the 12-month preperiod. The time-varying predictors were calculated based on utilization in the 6 month preceding each quarter. These preperiods are labelled „pre“ in the figure above

Figure 2 illustrates the complete study design. The outcome was repeatedly measured in the eight quarters of the follow-up period (month 13–36). The Elixhauser subscales and sociodemographic variables were calculated for the preperiod (month 1–12). All other predictors and the patient-centred networks were allowed to vary over time. In particular, we expected that provider networks can change and we assumed that how a patient was recently treated is the most informative to predict future hospitalizations. Hence, we determined for each quarter of the follow-up period, which providers were visited during the previous 6 months. These recently seen providers formed the basis for the patient-centred networks, although the tie strength between these providers was based on the number of patients shared from the psychiatric cohort during the corresponding calendar year/s.

The weights of ${FC}_{p}$ were determined based on the first year of the follow-up period. In the second year, we reused the obtained weights from the first year to calculate ${FC}_{p}$ and assessed the comparative model performance of a baseline model that only included control variables with models that added either ${FC}_{p}$ or ${C}_{p}$.

### Control variables

During the weight estimation and when assessing the influence of ${FC}_{p}$ or ${C}_{p}$ on hospital admissions, we adjusted for age, sex and the urbanization of the region of residency at hospital admission, of which the latter could be an enabling resource for utilization [30, 31] and most Elixhauser subscales during the preperiod [29]. The Elixhauser subscales describe binary comorbidity indicators derived from ICD-10 diagnoses. The urbanization of the region was derived from a classification of the Federal Office for Building and Regional Planning [32]. The classification categorizes each NUTS 3 region [33] of Germany into one of four categories: major city, urban area, rural area with urbanization tendencies and rural area (we combined the latter two due to the low prevalence of the rural area category). We also considered care dependency according to the classification of the German long-term care insurance, which was also documented in the data set. Until 2017, there were care levels ranging from 0 (low) to 3 (high) that reflect the need for support in activities of daily living (ADL). Level 3 was further divided into level 3 and level 3 (hardship case), and we labelled the latter level 4. The verification and assessment of care level is performed by the medical service of the health insurance funds according to a standardized procedure. We determined the proportion of the preperiod in which patients had a particular care level between 0 to 4. Moreover, we included antipsychotic prescriptions, previous hospitalizations due to schizophrenia or related comorbidities and contacts to psychiatrists, psychotherapists and neurologists in the previous two quarter as time-varying control variables (see supplement for additional details).

### Statistical methods

We employed generalized linear mixed models (GLMMs) for binary data to predict quarterly hospital admissions. We included a subject-specific random-intercept to account for the dependence of multiple observations per subject. For the first year of the follow-up, we only assessed one model to determine the weights of $F{C}_{p}$ by including the time-varying dummy indicators for the $\mathrm{k}$ different connection type specific sums ${s}_{j}/({n}_{p}({n}_{p}-1)/2)$ and control variables. (Please note: In a network without any ties, all sum *s*_*j*_ would be 0. Therefore, FCD would be equivalent to the intercept of the model. Care Density is undefined for patients without health care utilization, but FCD automatically estimates a base risk for these patients.)

For the second year, we compared multiple GLMMs. We compared a baseline model that only included control variables with models that added either ${C}_{p}$ or $F{C}_{p}$. ${FC}_{p}$ was calculated based on Eq. 5 and the weights from the first year. Given that we wanted ${FC}_{p}$ to only incorporate network information, we concealed the information on control variables by setting all control variables to their mean. In the model containing ${C}_{p}$, we included a dummy indicator that encodes whether patients were treated by one or fewer providers in the previous two quarters, because ${C}_{p}$ cannot be calculated in this case.

All models were estimated in SAS, version 9.4 (SAS Institute Inc.). We employed likelihood ratio tests, AIC and BIC to compare the models.

## Results

### Sample characteristics

We identified *N* = 21,016 patients with schizophrenia. On average patients were 53 years old (SD = 14.93) and 49.5% were female. Regarding common comorbidities, we found that 44.0% were diagnosed with depression and 14.6% with substance abuse disorder. The descriptive statistics of all other control variables are reported for the 12-month preperiod in Supplemental table 2. To illustrate that ${C}_{p}$ would overemphasize connections between GPs, we provide stacked barplots (Supplemental Fig. 2) that depict how much each connection type contributes to the total number of shared patients. We found that more than 55% of all patients were shared between GPs. Furthermore, we provide Lorenz curves stratified by connection type (Supplemental Fig. 3) that underscore the inequality in the distribution of shared patients due to outliers. The latter issue is particularly pronounced for same specialty connections. Supplemental Table 3 shows the proportion of patients with a certain connection type – irrespective of the strength of the connection – and the hospitalization rate for each quarter of the follow-up period. Connections were determined based on provider visits in the 6 month before each quarter. The most common connections were between GPs (26–28%), GPs and psychiatrists (19–20%) and between GPs and neurologists/psychiatrists (21–25%). However, we also found that a large part (between 35–38%) of the sample did not visit at least two relevant providers. The hospitalization rate reached 10% in the first quarter and varied between 6–7% for the remaining quarters.

**Fig. 3:**
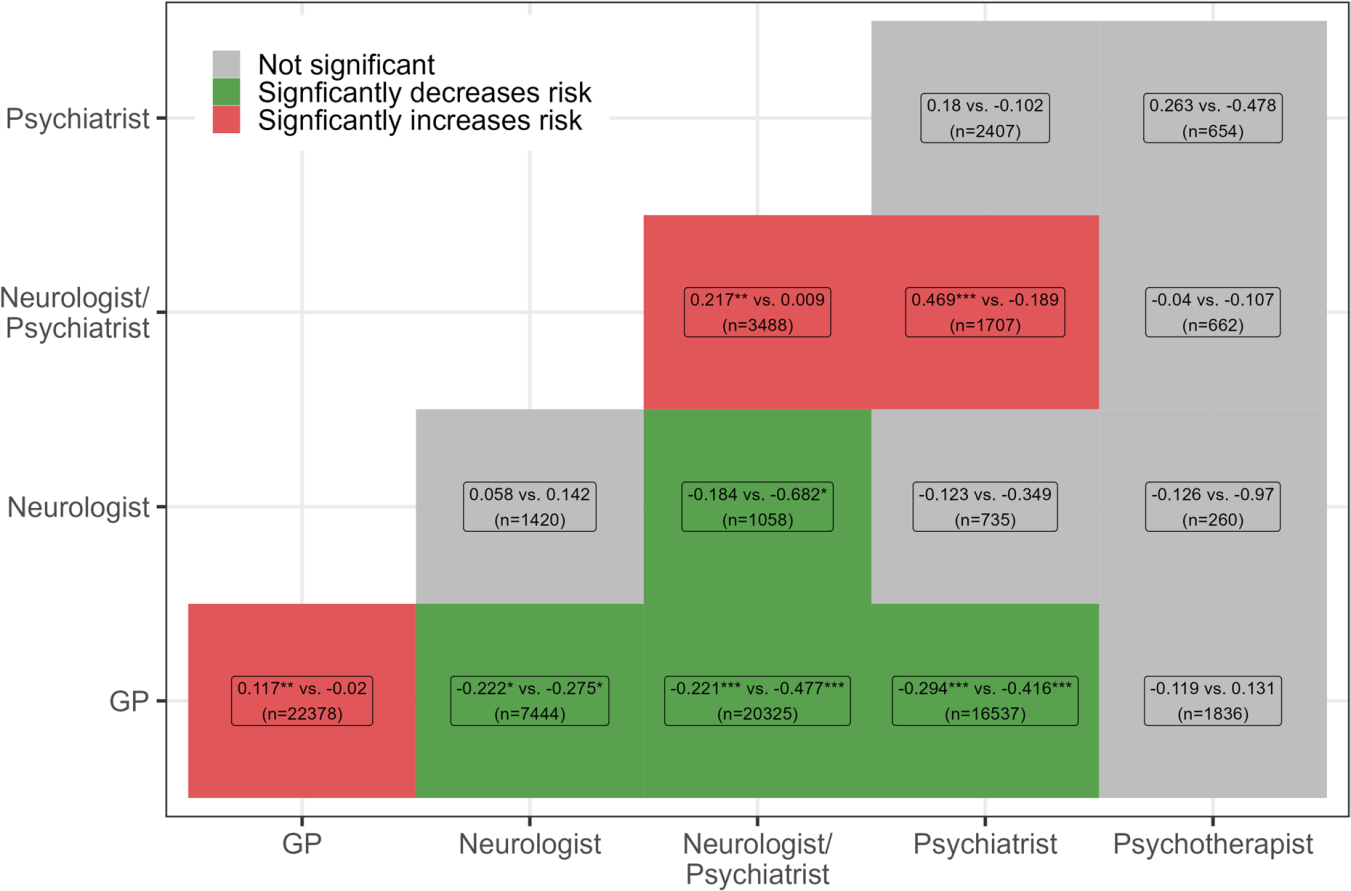
Heatmap comparing the weak and strong links by connection type *Notes*: n is the number of valid connections of that type with ≥ 3 shared patients, each segment displays both the FCD weight for the weak (left) and strong (right) link of that connection type. Weak links apply to connections that fall into the first tertile in terms of the number of shared patients, whereas strong links fall into the third tertile. The weights are positive / negative if the presence of that link in a patient-centered networks is associated with increased / decreased hospitalization rates compared to networks that do not contain this connection type

### Weight estimation and exploratory analysis of the ${{\varvec{F}}{\varvec{C}}}_{{\varvec{p}}}$ weights

The results of the GLMM for the first year can be found in the supplemental Tables 4a-c. Here, we focus on the selected coefficients that are based on network information. As explained in the methods sections, we estimated three weights for each connection type that compare the relative risk of a hospitalization in the reference category (i.e. the network does not contain this connection type) with the risk of a patient whose connections fall into the first (weak link), second (moderate link), or third tertile (strong link) in terms of the sum ${s}_{j}/({n}_{p}({n}_{p}-1)/2)$. The weights indicate whether these connections increase (positive coefficients) or decrease (negative coefficients) hospitalization risk. Moreover, a significant difference between weak and strong links indicates that the strength of the connection between the provider types influences hospitalization risk, which signifies that the coordination between the respective provider types may be particularly important when treating schizophrenia. Figure 3 displays a heatmap comparing the weights for the weak and strong links.

Having a weak link between GPs, between neurologist/psychiatrists or between neurologist/psychiatrist and psychiatrists significantly increased hospitalization risk compared to having no connection of that kind. Conversely, the connections between neurologist/psychiatrist and GPs reduce hospitalization risk relative to no link, albeit a weak link has less of a decreasing effect β = -0.22 (95% CI -0.33, -0.11) than a strong link β = -0.47 (95% CI -0.60, -0.35). A similar effect was observed for connections between GPs and psychiatrists, although the difference between the weak links, β = -0.29 (95% CI -0.41, -0.18), and strong links, β = -0.42 (95% CI -0.56, -0.27) is descriptively smaller. The complete list of coefficients with confidence bands can be found in Supplemental Table 4.

### Model comparison when predicting hospital admissions

Table 1 reports the results of the three GLMMs for the second year. It compares the baseline model to models that added either ${C}_{p}$ or ${FC}_{p}$. All non-significant control variables were omitted, but we report the complete list of coefficients in Supplemental Table 5a-b. The likelihood ratio test showed a significant improvement due to the inclusion of both ${C}_{p}$, Χ^2^[2] = 10.43, *p* < 0.01, and ${FC}_{p}$, Χ^2^ (1) = 53.30, *p* < 0.001. However, ${C}_{p}$ itself had no significant influence on hospital admissions. Instead, we found that the model fit increased due to the inclusion of a dummy indicator that encodes whether care density could not be calculated – i.e. whether a patient was treated by only one or zero outpatient providers. For ${FC}_{p}$, we found a significant influence on hospitalization risk, β = 0.61 [ 0.44; 0.78].

**Table 1:** Results of the generalized linear mixed model (second year)

	**Variable**	**Model 1 (baseline)**	**Model 2** **(incl. CD)**	**Model 3** **(incl. FCD)**
Elixhauser-subscales	Congestive heart failure	0.14*	0.15*	0.16*
	Cardiac arrythmias	0.14*	0.15**	0.14*
	Valvular disease	-0.29*	-0.31**	-0.36**
	Vascular disorders	0.28***	0.28***	0.32***
	Complicated hypertension	-0.15	-0.15	-0.19*
	Pulmonary diseases	0.10**	0.12**	0.11*
	Complicated Diabetes	0.13*	0.13	0.14*
	Renal failure	-0.14	-0.14	-0.19*
	Fluid and electrolyte	0.43***	0.46***	0.45***
	Alcohol Abuse	0.42***	0.45***	0.45***
	Drug Abuse	0.50***	0.55***	0.52***
	Depression	-0.11**	-0.11**	-0.10**
Other control variables	Hospitalization	1.67***	1.64***	1.62***
	Antipsychotics	0.35***	0.40***	0.39***
	Care level 0	0.37***	0.38***	0.40***
	Care level 1	0.17**	0.18**	0.19**
	Nursing home	0.18	0.18	0.22*
	Age	-0.02***	-0.02***	-0.02***
	Neurologist contacts	-0.02**	-0.01**	-0.01*
Coordination metrics	Care density		0.00	
	Care density dummy		0.10**	
	Fragmented Care Density			0.61***
Other model parameters	Intercept	-2.85***	-3.11***	-1.31***
	Patient-level variance	0.44***	0.42***	0.42***
Global Fit Statistics	-2 Log Likelihood	34,590.69	34,580.26	34,537.39
	AIC	34,680.69	34,674.26	34,629.39
	BIC	35,038.57	35,048.05	34,995.23

*p < .05, ***p < .01, ***p < .001, Cells contain estimated regression coefficients if not stated otherwise. Care density dummy is a dummy indicator that encodes whether care density can be calculated (i.e. whether the patient was treated by at least two outpatient providers). All non-significant control variables were omitted from this table. The full table is provided in the supplement

**Fig. 4:**
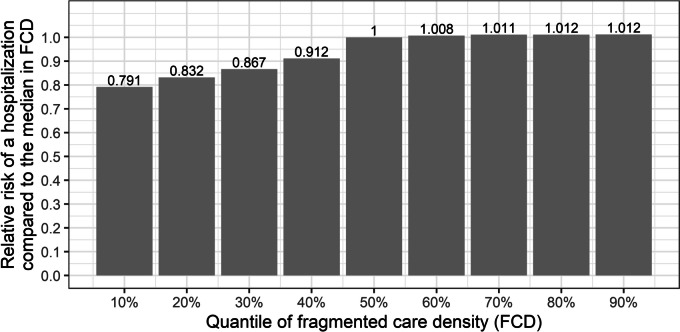
Relative Risk of a hospitalization dependent on the quantile in ${FC}_{p}$ compared to the median in ${FC}_{p}$ *Notes*: We calculated the deciles of FCD during the second year of the follow-up. The figure displays the predictions for the expected hospitalization risk based on these values relative to the risk of patients with median values in FCD. The predictions were made using model 3 with all control variables set to their mean (see Supplemental Table 5 for additional details on model 3).

To translate these coefficients to an illustrative scale, we predicted the relative risk of patients with a realistic range of values for ${FC}_{p}$. To that end, we predicted the hospitalization risk for patients with the median ${FC}_{p}$. Subsequently, we determined the relative risk for patients with other deciles in ${FC}_{p}$. Notably, the control variables were set to the mean during this prediction. The predictions are displayed in Fig. 4. We found only small and negligible differences in the hospitalization risk across patients with ${FC}_{p}$ values that exceed the median. However, we observe larger differences between patients below the median with a 21% decreased risk for patients at the first decile in ${FC}_{p}$.

## Discussion

In this paper, we proposed an extension of care density (CD) [11] called fragmented care density (FCD). Both metrics aim to measure care coordination based on patient-sharing networks. They are both suited for claims data analysis and they consider the number of shared patients between physicians, but they differ in terms of flexibility. While CD can be calculated uniformly across studies, FCD can be optimized for specific disorders, countries and outcomes because it allows connections between different types of providers to have varying and mutable weights. As a result, FCD is – in contrast to CD – not primarily affected by connections between provider types with a high patient volume, but instead emphasizes connections that predict the outcome of interest. Moreover, FCD can account for control variables, outliers and a potential non-linear relationship between shared patients and the outcome. These are desirable properties because it is well-known that the observed number of shared patients is prone to outliers and influenced by factors besides client-related coordination (e.g. the insurer, patient volume, regional proximity) [22, 34]. To prove that these advantages enhance model performance, we compared both metrics in their ability to explain quarterly hospital admissions due to or related to schizophrenia after accounting for other predictors that are known to be important. We found that CD had no significant influence on hospital admissions, whereas patients with lower deciles in FCD had a significantly and up to 21% lower risk when compared to those with median or higher values in FCD.

The relative hospitalization risks associated with various levels in FCD indicate that the quality of care coordination in the outpatient sector as measured by FCD has a meaningful but relatively small influence on future hospital admissions. In part, this small effect could reflect that schizophrenia is – at least during episodes of acute psychosis – not manageable in standard outpatient care. Instead, the literature suggests that the reduction of hospitalizations of patients with severe mental disorders requires more intensive forms of community-based care (e.g. additional case management or even outreaching multidisciplinary teams with a small case load [35–37]). Consistently, we found that within the 6 month prior to each quarter of the 24-month follow-up period, at least 35% of the assessed sample had fewer than two distinct outpatient providers. This finding supports the idea that most patients were not mainly treated in the outpatient sector. It also may explain why we found almost no difference in the absolute value of FCD between patients with median or higher values in FCD, because a metric based solely on connections between outpatient providers cannot vary for patients who had no or few relevant outpatient visits.

The assessment of the individual weights of FCD enables the researcher to validate that the metric correctly incorporates clinically relevant connections. Considering that FCD was set up as a risk score for future hospitalizations, it ought to assign negative weights to connections that decrease and positive weights to connections that increase hospitalization risk. The national guidelines on schizophrenia [38] emphasize the importance of an overarching cooperation between the GP and a psychiatrist for an effective treatment. Thus, it is plausible that connections between general practitioners (GPs) and psychiatrists or neurologist/psychiatrists significantly decreased FCD. In contrast, we expected that the weak links between GPs or between neurologist/psychiatrists would increase FCD, because multiple providers of the same or a similar discipline might signal dissatisfaction and a lack of continuity of care. It is well-known that a strong and reliable patient-provider-relationship improves patient outcomes and satisfaction [39], which might be missing for patients in sparsely connected networks with similar provider types. Interestingly, networks with strongly connected providers of the same discipline decreased FCD, which suggests that dispersion of visits across multiple providers does not hinder effective treatment if the involved providers share many patients (e.g. due to a shared office).

Other relevant predictors include known risk factors such as previous hospitalizations [40] or care dependency as well as comorbid substance abuse disorders [41]. At first glance, it may seem inconsistent that the prescriptions of antipsychotics was a risk factor for future hospitalizations despite its protective effect against rehospitalisation in earlier studies [42, 43]. However, considering that our selection criteria did not require a recent hospital stay, we presumably included a larger percentage of patients in remission, who are less likely to be rehospitalized than just recently released patients on medication.

### Generalizability and areas of application

Even though the FCD approach was developed based on German claims data, it should be applicable to other countries and disorders due to the estimation of context specific weights. Nevertheless, it should be noted that the validity of the approach depends on the assumption that shared patients suggest a true underlying relationship between physicians, which was only validated for the US market [12, 23]. However, given that studies based on patient-sharing networks were successfully conducted in the Netherlands, Canada, Italy and Australia [23], we assume that shared patients in claims data are related to frequent physician interactions, referrals and information exchange in many health care systems [44].

Regarding potential areas of application, we point out that care density and related patient-sharing metrics were successfully applied to predict all-cause or colon cancer specific mortality [45], the hospitalization risk of diabetic patients, cancer survivors and patients with congestive heart failure [11, 13, 14] and the prescription of overlapping benzodiazepine or interacting medications [46, 47]. It should be explored whether FCD can capture more nuanced patient-sharing patterns in these areas and improve predictions. Considering that simple coordination metrics (e.g. the usual provider of care index) tend to penalize large networks and dispersion of visits across multiple providers [48], we believe that the approach holds promise in areas were networks and coordination become complex and interdisciplinary.

### Limitations

It is still debated whether patient sharing or referrals networks [49] are better suited to explore coordination. In this study, we chose patient sharing networks as they are denser and include more connections between specialists. However, we are aware that the validity of relationships identified based on patient sharing information can vary between specialties [12] and the approach may not be ideal to identify ties to providers with a low patient volume (e.g. psychotherapists).

In addition, we focused solely on outpatient providers, although effective treatment of schizophrenia requires intersectoral coordination with hospitals and social services [50]. Unfortunately, we were not able to incorporate these additional sectors because statutory health insurance claims data does not contain information on the use of social services in Germany and hospital stays would be confounded by our optimization criterion (i.e. hospitalizations) for estimating the FCD weights.

We focused on a range of common psychological and psychiatric disorders to craft networks that capture collaboration among providers in the treatment of mental disorders. We decided to focus on a subset of diagnoses that require collaboration between providers, but the list of considered diagnoses does not include all potentially useful ones (e.g., it does not include personality disorders or proactive drug use). However, it is unlikely that the consideration of additional low-prevalent diagnoses would change the extracted networks substantially.

Moreover, we applied FCD only to one specific cohort of schizophrenia patients with an average age of 53. As the onset of schizophrenia typically occurs in the late teens to early thirties [51], a meaningful percentage of the sample might have been in remission. To further validate FCD, it should be applied to different samples and psychiatric conditions. Regarding general limitations of patient-sharing networks, we point out that coordination often leaves no formally documented trace. Most providers tend to use the telephone for client-related cooperation and observable financial reimbursements for cooperation and coordination remain an exception [52]. Thus, although patient-sharing networks represent an option to study patient streams that result from either good or bad coordination, researchers should keep in mind that metrics based on these networks remain surrogate variables that may lack pertinent aspects. In addition, they are affected by factors besides coordination. Relevant confounders include provider-characteristics (e.g. patient volume), area-level effects (e.g. population and physician density) and patient-characteristics (e.g. utilization patterns, clinical severity). While FCD can account for patient-level control variables during the weight estimation, it is not as straightforward to control for provider-level characteristics.

Lastly, we want to stress the importance of the market share of the data provider because it affects how long it takes to observe a sufficient amount of patient sharing. In the federal state Baden-Württemberg more than 39% of the population are insured by the statutory AOK [53] and German claims data are not by design restricted to a particular age group, employees of a certain company or a specific socioeconomic status. As a result, our population is more diverse than in Medicare or Medicaid datasets. We assume that populations with a high risk for chronic diseases will have denser networks than relatively healthy populations. In addition, a recent study found that networks obtained from Medicare data are more reliable than networks obtained from single private insurers [34].

Despite these limitations, we still advocate the use of network analytic techniques based on claims data because they are the most promising approach to understand coordination between providers during the treatment of patients with rare and complex disorders. The use of claims data enabled us to assess a long follow-up period of 2 years for a large sample of patients with schizophrenia. Moreover, the extensive data source made it easy to validate FCD on a separate year. High quality randomized controlled trials that assess the influence of improved coordination and collaboration do not exist for patients with schizophrenia [54] and are generally rarely financed. In addition, it is often infeasible to obtain self-reported information from all providers who are involved in the treatment.

## Conclusions

In this study, we introduced FCD, which is a flexible and transparent network measure, and used a cohort of patients with schizophrenia to demonstrate how FCD can facilitate our understanding of what type of specialties should be involved in the treatment of a particular illness, in order to reduce hospitalization risk. In addition, FCD can help us better understand which disciplines need to collaborate to provide optimal care and what types of provider constellations may hinder effective collaboration. Given that FCD can be adjusted for different disorders, health systems in different countries and alternative outcomes, FCD is broadly applicable. If FCD is validated further, it could be used to identify barriers and promoting factors that influence coordination. As a result, decision makers could adapt best practices more easily.

### Supplementary Information

**Additional file 1:** **Table 1.** Inclusion criteria for the number of shared patients. **Table 2. a** Descriptive statistics for the 12-month preperiod. **b** Descriptive statistics on the elixhauser subscales for the 12-month preperiod. **Table 3.** Hospitalization rate for each quarter in the follow-up period and the proportion of patients with a certain connection type within the 6 month prior to that quarter.** Table 4. a** Coefficients of the GLMM for binary data. **b** Coefficients of the GLMM for binary data (Eixhauser subscales). **c** Coefficients of the GLMM for binary data (${FC}_{p}$ weights). **Table 5.** **a** Coefficients of the GLMM for binary data (Elixhauser subscales). **b** Coefficients of the GLMM for binary data (all other predictors). **Figure 1.** The necessary steps to convert care density to fragmented care density. **Figure 2.** Stacked barplots on the distribution of the total number of shared patients among connection types. **Figure 3.** Lorenz curves for each connection type.

## Data Availability

In order to obtain access to the dataset, we requested the permission for these research questions and concluded a contract with the statutory health insurance AOK BW regarding data access. The study was approved by the data protection officer both at the AOK BW and our research institute. The received license permits the use of the data for the purpose of the research proposal within our company, exclusively. Data are owned by the German statutory health insurance AOK BW. To request the data please contact the institutional body of the AOK BW directly (Simon.Beuerle@bw.aok.de). In order to fulfill the legal requirements to obtain that kind of data, researchers must conclude a contract with the AOK BW regarding data access. The licensee is permitted to use the data for the purpose of the research proposal only. Licensees are not allowed to pass the data to a third party, or to create software or data bases with the exception of scientific publications.

## Abbreviations

CD: Care density
FCD: Fragmented care density
OCC: Outpatient care coordination
GP: General practitioner
GLMM: Generalized linear mixed models
AIC: Akaike information criterion
BIC: Bayesian information criterion

## References

[1] Murray CJ, Vos T, Lozano R, Naghavi M, Flaxman AD, Michaud C (2012). Disability-adjusted life years (DALYs) for 291 diseases and injuries in 21 regions, 1990–2010: a systematic analysis for the Global Burden of Disease Study 2010. The lancet.

[2] Keehan SP, Cuckler GA, Sisko AM, Madison AJ, Smith SD, Stone DA (2015). National health expenditure projections, 2014–24: spending growth faster than recent trends. Health Aff.

[3] Stange KC. The problem of fragmentation and the need for integrative solutions. Annals Family Med. 2009;7(2):100–3. 10.1370/afm.971 PMC2653966 19273863

[4] Cebul RD, Rebitzer JB, Taylor LJ, Votruba ME (2008). Organizational fragmentation and care quality in the US healthcare system. J Econom Perspectives.

[5] Peikes D, Chen A, Schore J, Brown R (2009). Effects of care coordination on hospitalization, quality of care, and health care expenditures among Medicare beneficiaries: 15 randomized trials. JAMA.

[6] Kocher RP, Adashi EY (2011). Hospital readmissions and the affordable care act: paying for coordinated quality care. JAMA.

[7] McWilliams JM (2016). Cost containment and the convenient tale of care coordination. N Engl J Med.

[8] Brown RS, Peikes D, Peterson G, Schore J, Razafindrakoto CM (2012). Six features of Medicare coordinated care demonstration programs that cut hospital admissions of high-risk patients. Health Aff.

[9] Kaufman BG, Spivack BS, Stearns SC, Song PH, O’Brien EC (2019). Review impact of accountable care organizations on utilization, care, and outcomes: a systematic review. Medical Care Research.

[10] Sinaiko AD, Landrum MB, Meyers DJ, Alidina S, Maeng DD, Friedberg MW (2017). Synthesis of research on patient-centered medical homes brings systematic differences into relief. Health Aff.

[11] Pollack CE, Weissman GE, Lemke KW, Hussey PS, Weiner JP (2013). Patient sharing among physicians and costs of care: a network analytic approach to care coordination using claims data. J Gen Intern Med.

[12] Barnett ML, Landon BE, O’malley AJ, Keating NL, Christakis NA (2011). Mapping physician networks with self-reported and administrative data. Health Services Res.

[13] Pollack CE, Frick KD, Herbert RJ, Blackford AL, Neville BA, Wolff AC (2014). It’s who you know: patient-sharing, quality, and costs of cancer survivorship care. J Cancer Surviv.

[14] Pollack CE, Lemke KW, Roberts E, Weiner JP (2015). Patient sharing and quality of care: measuring outcomes of care coordination using claims data. Med Care.

[15] Härter M, Klesse C, Bermejo I, Schneider F, Berger M. Unipolar depression: diagnostic and therapeutic recommendations from the current S3/National Clinical Practice Guideline. Dtsch Arztebl Int. 2010;107(40):700. 10.3238/arztebl.2010.0700 PMC2965372 21031129

[16] Wiersma D, Wanderling J, Dragomirecka E, Ganev K, Harrison G, Der Heiden WA (2000). Social disability in schizophrenia: its development and prediction over 15 years in incidence cohorts in six European centres. Psychol Med.

[17] Möller H-J, Jäger M, Riedel M, Obermeier M, Strauss A, Bottlender R (2010). The Munich 15-year follow-up study (MUFUSSAD) on first-hospitalized patients with schizophrenic or affective disorders: comparison of psychopathological and psychosocial course and outcome and prediction of chronicity. European archives of psychiatry.

[18] Frey S (2014). The economic burden of schizophrenia in Germany: a population-based retrospective cohort study using genetic matching. Eur Psychiatry.

[19] Adair CE, McDougall GM, Mitton CR, Joyce AS, Wild TC, Gordon A (2005). Continuity of care and health outcomes among persons with severe mental illness. Psychiatr Serv.

[20] Erler A, Beyer M, Muth C, Gerlach F, Brennecke R (2009). Garbage in-garbage out? Validity of coded diagnoses from GP claims records. Das Gesundheitswesen.

[21] Klauber J, Günster C, Gerste B, Robra B-P, Schmacke N. Versorgungs-Report 2013/2014: Schwerpunkt: Depression. Stuttgart: Schattauer Verlag; 2014.

[22] Landon BE, Keating NL, Barnett ML, Onnela J-P, Paul S, O’Malley AJ (2012). Variation in patient-sharing networks of physicians across the United States. JAMA.

[23] DuGoff EH, Fernandes-Taylor S, Weissman GE, Huntley JH, Pollack CE (2018). A scoping review of patient-sharing network studies using administrative data. Translational behavioral medicine.

[24] Gao J, Moran E, Li Y-F, Almenoff PL. Predicting potentially avoidable hospitalizations. Med Care. 2014;52(2):164–71. 10.1097/MLR.0000000000000041 24374413

[25] Jee SH (2006). Cabana MDJMCR, review. Indices Continuity Care: A System Rev Literature.

[26] Gühne U, Weinmann S, Riedel-Heller S, Becker T, Aderhold V, Bechdolf A. Kurzfassung der S3-Leitlinie Psychosoziale Therapien bei schweren psychischen Erkrankungen: Deutsche Gesellschaft für Psychiatrie und Psychotherapie, Psychosomatik und Nervenheilkunde. 2019. https://www.dgppn.de/_Resources/Persistent/4a081f97b24d101a36bd970d5fd3823d562404cd/S3-LL-PsychosozTherapien-Kurzfassung.pdf. Accessed 5 Dec 2023.

[27] Falkai P, Hasan A. Praxishandbuch Schizophrenie: Diagnostik-Therapie-Versorgungsstrukturen. München: Urban & Fischer/Elsevier; 2019.

[28] Buckley PF, Miller BJ, Lehrer DS, Castle DJ (2009). Psychiatric comorbidities and schizophrenia. Schizophr Bull.

[29] Quan H, Sundararajan V, Halfon P, Fong A, Burnand B, Luthi J-C, et al. Coding algorithms for defining comorbidities in ICD-9-CM and ICD-10 administrative data. Med Care. 2005;43(11):1130–39. 10.1097/01.mlr.0000182534.19832.83 16224307

[30] Andersen RM. Revisiting the behavioral model and access to medical care: does it matter? J Health Soc Behav. 1995;36(1):1–10. 7738325

[31] Lin Y-J, Tian W-H, Chen C-C (2011). Urbanization and the utilization of outpatient services under National Health Insurance in Taiwan. Health Policy.

[32] Bundesinstitut für Bau-, Stadt- und Raumforschung. Laufende Raumbeobachtung - Raumabgrenzungen. https://www.bbsr.bund.de/BBSR/DE/forschung/raumbeobachtung/Raumabgrenzungen/deutschland/kreise/siedlungsstrukturelle-kreistypen/kreistypen.html. Accessed 2 Oct 2023.

[33] Eurostat. NUTS - Nomenclature of territorial units for statistics. 2021. https://ec.europa.eu/eurostat/web/nuts/background. Accessed 2 Oct 2023.

[34] Trogdon JG, Weir W, Shai S, Mucha P, Kuo T, Meyer A (2019). Comparing shared patient networks across payers. J Gen Intern Med.

[35] Karow A, Reimer J, König HH, Heider D, Bock T, Huber C, et al. Cost-effectiveness of 12-month therapeutic assertive community treatment as part of integrated care versus standard care in patients with schizophrenia treated with quetiapine immediate release (ACCESS trial). J Clin Psychiatry. 2012;73(3):402–8. 10.4088/JCP.11m06875 22490266

[36] Malone D, Marriott SV, Newton‐Howes G, Simmonds S, Tyrer P. Community mental health teams (CMHTs) for people with severe mental illnesses and disordered personality. Cochrane Database Syst Rev. 2007. 10.1002/14651858.CD000270.pub2. 10.1002/14651858.CD000270.pub2 PMC4171962 17636625

[37] Dieterich M, Irving CB, Bergman H, Khokhar MA, Park B, Marshall M. Intensive case management for severe mental illness. Cochrane Database Syst Rev. 2017; 10.1002/14651858.CD007906.pub3. 10.1002/14651858.CD007906.pub3 PMC6472672 28067944

[38] Gaebel W, Hasan A, Falkai P. S3-Leitlinie Schizophrenie. Berlin: Springer-Verlag; 2019.

[39] Van Walraven C, Oake N, Jennings A, Forster AJ (2010). The association between continuity of care and outcomes: a systematic and critical review. J Eval Clin Pract.

[40] San L, Bernardo M, Gómez A, Peña M (2013). Factors associated with relapse in patients with schizophrenia. Int J Psychiatry Clin Pract.

[41] Weiser M, Knobler HY, Noy S, Kaplan Z (2002). Clinical characteristics of adolescents later hospitalized for schizophrenia. Am J Med Genet.

[42] Bodén R, Brandt L, Kieler H, Andersen M, Reutfors J (2011). Early non-adherence to medication and other risk factors for rehospitalization in schizophrenia and schizoaffective disorder. Schizophr Res.

[43] Mgutshini T (2010). Risk factors for psychiatric re-hospitalization: An exploration. Int J Ment Health Nurs.

[44] Uddin S, Kelaher M, Srinivasan U (2016). A framework for administrative claim data to explore healthcare coordination and collaboration. Aust Health Rev.

[45] Hussain T, Chang H-Y, Veenstra CM, Pollack CE (2015). Collaboration between surgeons and medical oncologists and outcomes for patients with stage III colon cancer. Journal of oncology practice.

[46] Ong M-S, Olson KL, Cami A, Liu C, Tian F, Selvam N, et al. Provider patient-sharing networks and multiple-provider prescribing of benzodiazepines. J Gen Intern Med. 2016;31(2):164–71. 10.1007/s11606-015-3470-8 PMC4720655 26187583

[47] Ong M-S, Olson KL, Chadwick L, Liu C, Mandl KD (2017). The impact of provider networks on the co-prescriptions of interacting drugs: a claims-based analysis. Drug Saf.

[48] Jee SH, Cabana MD (2006). Indices for continuity of care: a systematic review of the literature. Medical Care Research Review.

[49] Mascia D, Angeli F, Vincenzo Di F (2015). Medicine effect of hospital referral networks on patient readmissions. Soc Scie.

[50] Schmidt-Kraepelin C, Janssen B, Gaebel W (2009). Prevent Rehospitalization Schizophrenia: Results Integrated Care Project Germany. Eur Arch Psychiatry Clin Neurosci.

[51] Immonen J, Jääskeläinen E, Korpela H, Miettunen J (2017). Age at onset and the outcomes of schizophrenia: A systematic review and meta-analysis. Early Interv Psychiatry.

[52] Bramesfeld A, Ungewitter C, Böttger D, El Jurdi J, Losert C, Kilian R (2012). What promotes and inhibits cooperation in mental health care across disciplines, services and service sectors? A qualitative study. Epidemiology and psychiatric sciences.

[53] AOK-Bundesverband. Zahlen und Fakten. 2020. https://aok-bv.de/imperia/md/aokbv/aok/zahlen/zuf_2020_web.pdf. Accessed 3 Apr 2021.

[54] Reilly S, Planner C, Gask L, Hann M, Knowles S, Druss B, et al. Collaborative care approaches for people with severe mental illness. Cochrane Database Syst Rev. 2013. 10.1002/14651858.CD009531.pub2. 10.1002/14651858.CD009531.pub2 24190251

