# Supplementary material for: A flexible approach to measure care coordination based on patient-sharing networks
Source: BMC Med Res Methodol. 2024 Jan 3;24:1. doi: 10.1186/s12874-023-02106-0 (PMC10762822; doi:10.1186/s12874-023-02106-0)
Supplement: Supplementary file 1 — Additional file 1: Table 1. Inclusion criteria for the number of shared patients. Table 2. a Descriptive statistics for the 12-month preperiod. b Descriptive statistics on the elixhauser subscales for the 12-month preperiod. Table 3. Hospitalization rate for each quarter in the follow-up period and the proportion of patients with a certain connection type within the 6 month prior to that quarter. Table 4. a Coefficients of the GLMM for binary data. b Coefficients of the GLMM for binary data (Eixhauser subscales). c Coefficients of the GLMM for binary data (${FC}_{p}$FCp weights). Table 5. a Coefficients of the GLMM for binary data (Elixhauser subscales). b Coefficients of the GLMM for binary data (all other predictors). Figure 1. The necessary steps to convert care density to fragmented care density. Figure 2. Stacked barplots on the distribution of the total number of shared patients among connection types. Figure 3. Lorenz curves for each connection type. [file 12874_2023_2106_MOESM1_ESM.docx]

# **Supplementary Material**

## **Inclusion criteria for the psychiatric cohort**

| **Table 1: Inclusion criteria for the number of shared patients** |
| --- |
| 1. Age ≥ 18 years |
| 1. Continously insured by the AOK BW |
| 1. Place of residency in Baden-Württemberg (southwestern Germany) |
| 1. Diagnosis in the respective calender year due to alcohol abuse (F10.x), schizophrenia (F20.x), bipolar disorder (F31.x), depressive episode (F32.x), recurrent depressive disorder (F33.x), dysthymia (F34.1), phobic anxiety disorder (F40.x), other anxiety disorders (F41.x), adjustment disorder (F43.2), or somatoform disorder (F45.x). Each diagnosis had to be documented by a hospital, a psychiatric or university outpatient clinic, a mental health specialist or in two successive quarters. |

AOK is a large statutory health insurance company in Germany, BW Baden-Württemberg (region in southwestern Germany)

**Figure 1: The necessary steps to convert care density to fragmented care density.**


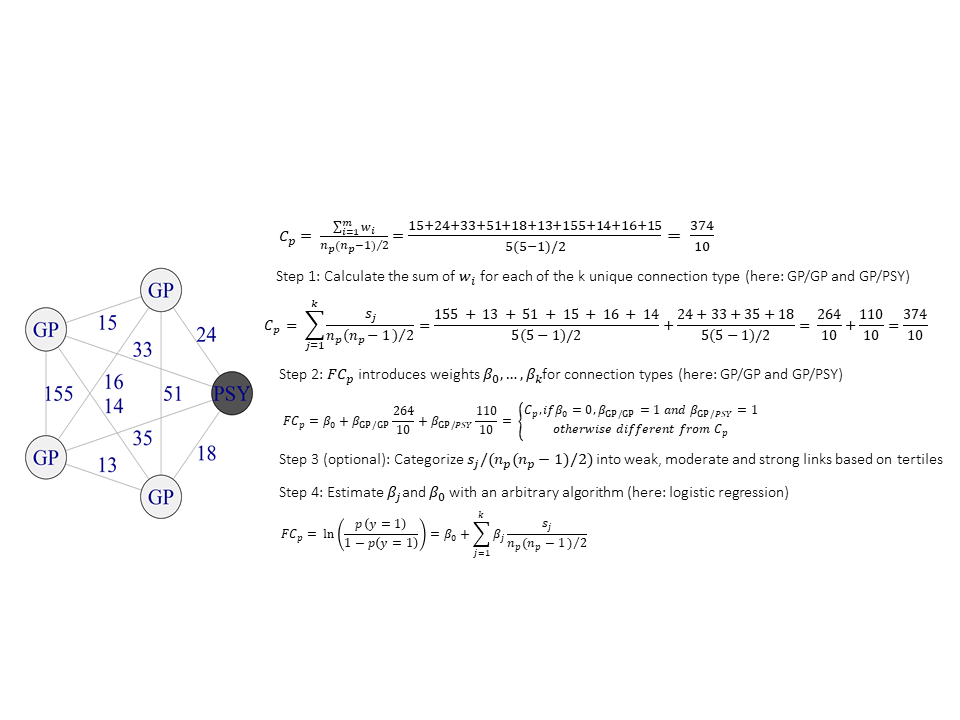


Notes: GP General practitioner, PSY psychiatrist, FCD fragmented care density, CD care density, we used

the patient-centered network of patient B from figure 1 as an example to illustrate the calculation of CD and FCD

## **Descriptive statistics**

**Table 2a descriptive statistics for the 12-month preperiod**

| **Category** | **Variable** | **Mean (SD)** | **Median (IQR)** | | **Frequency (%)** |
| --- | --- | --- | --- | --- | --- |
| Healthcare service use | Hospitalization (reference=none) |  |  | 4883 (23.23%) | |
|  | Care level 0 | 0.06 (0.23) | 0 (0) |  | |
|  | Care level 1 | 0.1 (0.3) | 0 (0) |  | |
|  | Care level 2 | 0.04 (0.2) | 0 (0) |  | |
|  | Care level 3 | 0.01 (0.11) | 0 (0) |  | |
|  | Care level 4 | 0 (0.02) | 0 (0) |  | |
|  | Nursing home | 0.05 (0.2) | 0 (0) |  | |
|  | Prescription of antipsychotics | 0.85 (0.36) | 1 (0) |  | |
| Socio-demographic variables | Residency in major city |  |  | 4531 (21.56%) | |
|  | Residency in urban area |  |  | 13425 (63.88%) | |
|  | Residency in rural area |  |  | 3060 (14.56%) | |
|  | Age | 53.36 (14.93) | 53 (20) |  | |
|  | Sex (reference=female) |  |  | 10395 (49.46%) | |
| Follow up visits | Neurologist | 0.94 (3.21) | 0 (0) | 0.94 (3.21%) | |
|  | Psychiatrist | 4.97 (8.75) | 3 (8) | 4.97 (8.75%) | |
|  | Psychotherapist | 0.56 (4.93) | 0 (0) | 0.56 (4.93%) | |

*Notes:* SD standard deviation, IQR interquartile range, all numbers were rounded to 2 decimal places, for all variables with binary coding 1 indicates that the condition or status was present, care levels as well as nursing home care are considered as the proportion of the pre-period in which patients were categorized as level 0, 1, 2, 3, 4 or as a patient receiving nursing home care.

| **Table 2b descriptive statistics on the elixhauser subscales for the 12-month preperiod** | | | | | | |  |
| --- | --- | --- | --- | --- | --- | --- | --- |
| **Category** | **Variable** | **Frequency (%)** | |  |  |  | |
| Elixhauser  (ICD-10)  (included) | Congestive heart failure | 1514 (7.2%) |  | |  |  | |
|  | Cardiac arrythmias | 1880 (8.95%) |  | |  |  | |
|  | Valvular disease | 569 (2.71%) |  | |  |  | |
|  | Pulomonary circulation disorders | 178 (0.85%) |  | |  |  | |
|  | Peripheral vascular disorders | 886 (4.22%) |  | |  |  | |
|  | Uncomplicated hypertension | 7370 (35.07%) |  | |  |  | |
|  | Complicated hypertension | 1248 (5.94%) |  | |  |  | |
|  | Paralysis | 545 (2.59%) |  | |  |  | |
|  | Neurodegenerative diseases | 1858 (8.84%) |  | |  |  | |
|  | Chronic pulmonary diseases | 4001 (19.04%) |  | |  |  | |
|  | Uncomplicated Diabetes | 4107 (19.54%) |  | |  |  | |
|  | Complicated Diabetes | 2229 (10.61%) |  | |  |  | |
|  | Hypothyroidism | 2854 (13.58%) |  | |  |  | |
|  | Renal failure | 1100 (5.23%) |  | |  |  | |
|  | Liver disease | 2170 (10.33%) |  | |  |  | |
|  | Solid tumor without metastasis | 809 (3.85%) |  | |  |  | |
|  | Rheumatoid arthritis | 726 (3.45%) |  | |  |  | |
|  | Coagulopathy | 377 (1.79%) |  | |  |  | |
|  | Obesity | 4728 (22.5%) |  | |  |  | |
|  | Weight loss | 432 (2.06%) |  | |  |  | |
|  | Fluid and electrolyte diseases | 1296 (6.17%) |  | |  |  | |
|  | Blood loss anemia | 106 (0.5%) |  | |  |  | |
|  | Deficiency anemia | 1025 (4.88%) |  | |  |  | |
|  | Alcohol Abuse | 2010 (9.56%) |  | |  |  | |
|  | Drug Abuse | 1569 (7.47%) |  | |  |  | |
|  | Depression | 9253 (44.03%) |  | |  |  | |
|  | Anxiety disorder | 3044 (14.48%) |  | |  |  | |
|  | Somatoforme Disorder | 3264 (15.53%) |  | |  |  | |
| Elixhauser  (ICD-10)  (excluded) | Peptic ulcer disease | 153 (0.73%) |  | |  |  | |
|  | Metastatic solid tumor | 119 (0.57%) |  | |  |  | |
|  | Malignancy (incl. lymphoma) | 65 (0.31%) |  | |  |  | |
|  | AIDS/HIV | 41 (0.2%) |  | |  |  | |

*Notes:* SD standard deviation, IQR interquartile range, all percentages were rounded to 2 decimal places, for all variables with binary coding 1 indicates that the condition or status was present

## **Additional information on the control variables**

Due to space restrictions, we reserved some additional information for the supplement. First, as mentioned in the article, we included most elixhauser subscales. The scales for hiv, peptic ulcer disease, metastatic cancer and lymphoma were excluded due to the low incidence rate of the corresponding conditions.

However, we controlled for psychological comorbidities more comprehensively than the elixhauser scales by assessing somatoform and anxiety disorders in the preperiod as well. The descriptive statistics for all comorbidities are shown in table 2b.

Second, we controlled for care dependency, because patients with schizophrenia can develop core “deficit” negative symptoms that are often enduring and may worsen over the longitudinal course of the illness. This can lead to the necessity to provide nursing home care for older patients and hospital and family care for younger patients [1]. Care dependency for all formal care recipients in Germany used to be categorized in one of three care levels based on the daily time required assistance in performing activities of daily living. Care level 1, 2, and 3 implied requiring basic care such as washing, feeding, or dressing for at least 0.75, 2, and 4 hours daily time, respectively [2]. Care level 0 is reserved for patients with dementia, mental disorders or mental disability and can also involve non-traditional services (e,g, providing stimulating activaties such as conversations or preparing a memory album) and care level 4 is reserved for cases with an exceptionally high care dependency who qualify for a hardship arrangement.

We controlled for the proportion of the pre-period in which patients were categorized as level 0, 1, 2, 3, 4 or as a patient receiving nursing home care.

Third, we did not directly control for the number of visits to a psychiatrists, psychotherapist or neurologist, but instead for the number of unique dates a service was billed by these specialists. However, since visits without billed services are extremely rare for specialist appointments, this proxy should be identical.

## **Illustrating the empirical pitfalls when assessing the number of shared patients**

Figure 2 illustrates how the total number of shared patients and the number of connections are distributed among the various connection types for each calender year. Connections between GPs clearly have the largest share. However, this is even more pronounced when assessing the number of shared patients as opposed to the number of connections due to their high patient volume. Connections between GPs make up between 57.3% and 57.8% with respect to the number of shared patients. The connections between GPs and specialists receive a rather low weight when calculating care density, because the metric places a high emphasize on the absolute number of shared patients.

**Figure 2: Stacked barplots on the distribution of the total number of shared patients among connection types**


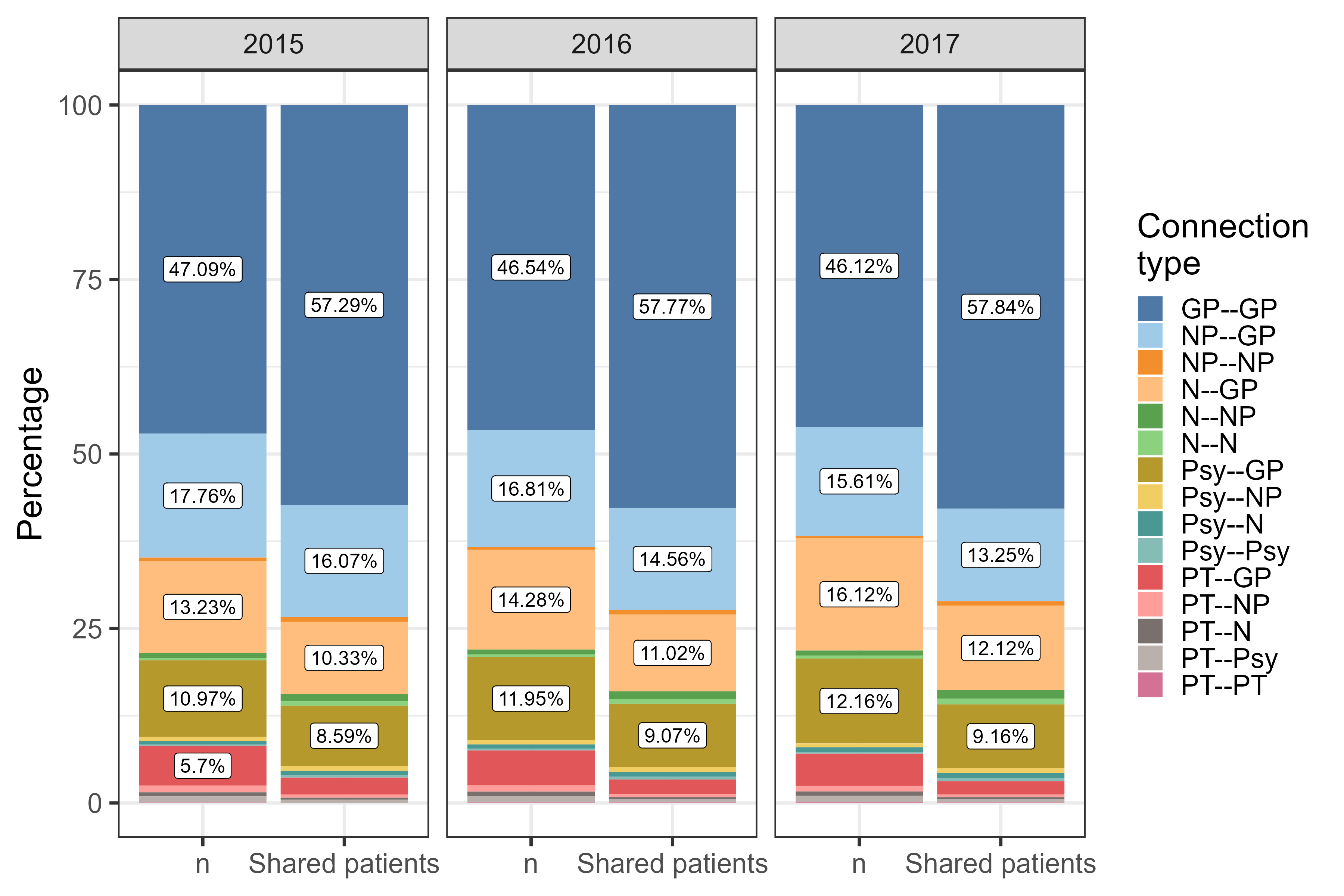


*Notes:* GP general practitioner, NP neurologist/psychiatrist, Psy psychiatrist, PT psychotherapist, N neurologist. Shared patients: Number of shared patients to which both providers bill their services, n: number of connections with at least 3 shared patients of particular connection type

Figure 3 depicts Lorenz curves to illustrates how the number of shared patients are distributed among the connections of a particular type. The Lorenz curve was originally developed to visualize the concentration of wealth [3], but it can more generally be used to visualize how a population’s sum of a variable is distributed among the people within the population. Perfect equality (i.e. each connection shares the same number of patients) would be depicted by a straight line. For some connection types, we observe that the top 10% of the connections share about 55-60% of all shared patients (e.g. GP – GP, NP – NP). This issue is pronounced for same specialty connections, which could indicate outliers due to collective practices.

**Figure 3: Lorenz curves for each connection type**


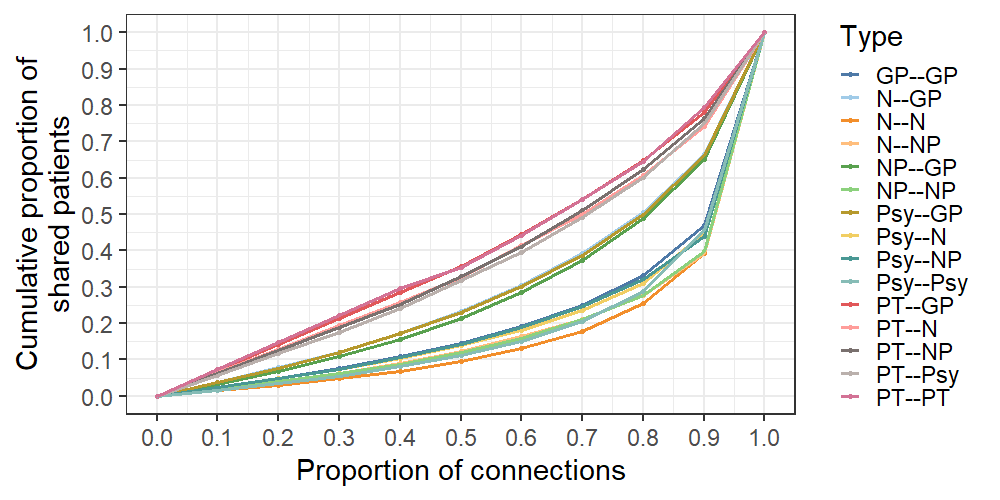


*Notes:* GP general practitioner, NP neurologist/psychiatrist, Psy psychiatrist, PT psychotherapist, N neurologist, n number of connections

**Table 3 hospitalization rate for each quarter in the follow-up period and the proportion of patients with a certain connection type within the 6 month prior to that quarter**

| **Category** | **Variable** | **Quarter of the 24-month follow-up period:** | | | | | | | |
| --- | --- | --- | --- | --- | --- | --- | --- | --- | --- |
|  |  | **Q1** | **Q2** | **Q3** | **Q4** | **Q5** | **Q6** | **Q7** | **Q8** |
|  | No connection | 0.37 | 0.35 | 0.35 | 0.36 | 0.37 | 0.38 | 0.38 | 0.37 |
| Connection Type | GP – GP | 0.26 | 0.26 | 0.26 | 0.28 | 0.28 | 0.26 | 0.26 | 0.27 |
|  | N – GP | 0.08 | 0.09 | 0.09 | 0.09 | 0.09 | 0.09 | 0.09 | 0.09 |
|  | N – N | 0.02 | 0.02 | 0.02 | 0.02 | 0.02 | 0.02 | 0.02 | 0.02 |
|  | N – NP | 0.01 | 0.01 | 0.01 | 0.01 | 0.01 | 0.01 | 0.01 | 0.01 |
|  | NP – GP | 0.23 | 0.25 | 0.25 | 0.23 | 0.22 | 0.22 | 0.22 | 0.21 |
|  | NP – NP | 0.04 | 0.04 | 0.04 | 0.04 | 0.04 | 0.04 | 0.04 | 0.04 |
|  | Psy – GP | 0.19 | 0.20 | 0.20 | 0.20 | 0.20 | 0.20 | 0.20 | 0.20 |
|  | Psy – N | 0.01 | 0.01 | 0.01 | 0.01 | 0.01 | 0.01 | 0.01 | 0.01 |
|  | Psy – NP | 0.02 | 0.02 | 0.02 | 0.02 | 0.02 | 0.02 | 0.02 | 0.02 |
|  | Psy – Psy | 0.03 | 0.03 | 0.03 | 0.03 | 0.03 | 0.03 | 0.03 | 0.03 |
|  | PT – GP | 0.02 | 0.02 | 0.02 | 0.02 | 0.02 | 0.02 | 0.02 | 0.02 |
|  | PT - N | 0.00 | 0.00 | 0.00 | 0.00 | 0.00 | 0.00 | 0.00 | 0.00 |
|  | PT – NP | 0.01 | 0.01 | 0.01 | 0.01 | 0.01 | 0.01 | 0.01 | 0.01 |
|  | PT – Psy | 0.01 | 0.01 | 0.01 | 0.01 | 0.01 | 0.01 | 0.01 | 0.01 |
| Outcome: | Hospitalization rate | 0.10 | 0.07 | 0.07 | 0.06 | 0.06 | 0.07 | 0.06 | 0.06 |
|  | Number hospitalized | 2122 | 1418 | 1401 | 1284 | 1277 | 1375 | 1311 | 1284 |

*Notes: N=21,016, Q quarter,* GP general practitioner, NP neurologist/psychiatrist, Psy psychiatrist, PT psychotherapist, all numbers were rounded to 2 decimal places, we only considered hospitalizations due to schizophrenia or a common comorbidity

## **Results of the GLMM to predict hospital admission in the first year of the follow-up:**

**Table 4a coefficients of the GLMM for binary data**

| **Category** | **Variable** | **Estimate** | **SE** | | **Statistic** | | **p** | | | **CI lower** | | **CI upper** | |
| --- | --- | --- | --- | --- | --- | --- | --- | --- | --- | --- | --- | --- | --- |
| Intercept |  | 1.87 | | 0.08 | | 23.61 | | 0.000 | 1.71 | | 2.02 | |  |
| Healthcare service and costs | Inpatient costs | -1.36 | | 0.03 | | -44.11 | | 0.000 | -1.42 | | -1.30 | |  |
|  | Prescription of antipsychotics | -0.01 | | 0.04 | | -0.35 | | 0.726 | -0.10 | | 0.07 | |  |
|  | Care level 0 | -0.37 | | 0.06 | | -6.48 | | 0.000 | -0.49 | | -0.26 | |  |
|  | Care level 1 | -0.23 | | 0.05 | | -4.29 | | 0.000 | -0.34 | | -0.13 | |  |
|  | Care level 2 | -0.02 | | 0.09 | | -0.22 | | 0.829 | -0.20 | | 0.16 | |  |
|  | Care level 3 | 0.10 | | 0.17 | | 0.62 | | 0.533 | -0.22 | | 0.43 | |  |
|  | Care level 4 | 1.40 | | 1.18 | | 1.19 | | 0.233 | -0.90 | | 3.70 | |  |
|  | Nursing home | -0.09 | | 0.09 | | -0.94 | | 0.347 | -0.27 | | 0.10 | |  |
| Socio-demographic variables | Residency in major city | 0.06 | | 0.05 | | 1.32 | | 0.186 | -0.03 | | 0.16 | |  |
|  | Residency in urban area | 0.02 | | 0.04 | | 0.43 | | 0.665 | -0.06 | | 0.10 | |  |
|  | Age | 0.02 | | 0.00 | | 18.00 | | 0.000 | 0.02 | | 0.02 | |  |
|  | Sex | -0.05 | | 0.03 | | -1.58 | | 0.114 | -0.11 | | 0.01 | |  |
| Follow up contacts | Neurologist | 0.01 | | 0.01 | | 1.76 | | 0.078 | 0.00 | | 0.02 | |  |
|  | Psychiatrist | 0.00 | | 0.00 | | -0.33 | | 0.740 | 0.00 | | 0.00 | |  |
|  | Psychotherapist | 0.00 | | 0.00 | | 0.02 | | 0.988 | 0.00 | | 0.00 | |  |

*Notes:* GLMM generalized linear mixed model, SE standard error, CI Confidence interval, all numbers were rounded to 2 decimal places (p values to 3 decimal places), care levels as well as nursing home care are considered as the proportion of the pre-period in which patients were categorized as level 0, 1, 2, 3, 4 or as a patient receiving nursing home care.

**Table 4b coefficients of the GLMM for binary data (Eixhauser subscales)**

| **Category** | **Variable** | **Estimate** | **SE** | | **Statistic** | | **p** | | **CI lower** | | **CI upper** | |
| --- | --- | --- | --- | --- | --- | --- | --- | --- | --- | --- | --- | --- |
| Elixhauser (ICD-10) (physio-logical) | Congestive heart failure | 0.13 | 0.07 | -1.91 | | 0.056 | | 0.00 | | 0.26 | |  |
|  | Cardiac arrythmias | 0.05 | 0.05 | -0.90 | | 0.370 | | -0.05 | | 0.15 | |  |
|  | Valvular disease | -0.03 | 0.10 | 0.28 | | 0.781 | | -0.22 | | 0.17 | |  |
|  | Pulomonary circulation disorders | -0.01 | 0.15 | 0.10 | | 0.919 | | -0.31 | | 0.28 | |  |
|  | Peripheral vascular disorders | 0.22 | 0.07 | -3.04 | | 0.002 | | 0.08 | | 0.37 | |  |
|  | Uncomplicated hypertension | -0.04 | 0.04 | 1.21 | | 0.227 | | -0.12 | | 0.03 | |  |
|  | Complicated hypertension | -0.15 | 0.07 | 2.06 | | 0.040 | | -0.30 | | -0.01 | |  |
|  | Paralysis | 0.05 | 0.09 | -0.58 | | 0.565 | | -0.13 | | 0.23 | |  |
|  | Neurodegenerative diseases | 0.04 | 0.05 | -0.80 | | 0.422 | | -0.06 | | 0.14 | |  |
|  | Chronic pulmonary diseases | 0.06 | 0.04 | -1.76 | | 0.079 | | -0.01 | | 0.14 | |  |
|  | Uncomplicated Diabetes | 0.03 | 0.05 | -0.52 | | 0.601 | | -0.07 | | 0.12 | |  |
|  | Complicated Diabetes | 0.13 | 0.06 | -2.07 | | 0.038 | | 0.01 | | 0.25 | |  |
|  | Hypothyroidism | 0.00 | 0.04 | -0.01 | | 0.995 | | -0.09 | | 0.09 | |  |
|  | Renal failure | 0.07 | 0.07 | -0.93 | | 0.354 | | -0.07 | | 0.21 | |  |
|  | Liver disease | -0.02 | 0.05 | 0.39 | | 0.696 | | -0.11 | | 0.08 | |  |
|  | Solid tumor without metastasis | 0.05 | 0.08 | -0.67 | | 0.502 | | -0.10 | | 0.21 | |  |
|  | Rheumatoid arthritis | -0.20 | 0.09 | 2.20 | | 0.028 | | -0.37 | | -0.02 | |  |
|  | Coagulopathy | 0.00 | 0.10 | 0.04 | | 0.965 | | -0.21 | | 0.20 | |  |
|  | Obesity | 0.02 | 0.04 | -0.46 | | 0.644 | | -0.06 | | 0.09 | |  |
|  | Weight loss | 0.13 | 0.09 | -1.40 | | 0.162 | | -0.05 | | 0.31 | |  |
|  | Fluid and electrolyte diseases | 0.52 | 0.05 | -9.69 | | 0.000 | | 0.41 | | 0.62 | |  |
|  | Blood loss anemia | -0.11 | 0.20 | 0.55 | | 0.581 | | -0.50 | | 0.28 | |  |
|  | Deficiency anemia | 0.10 | 0.06 | -1.53 | | 0.127 | | -0.03 | | 0.22 | |  |
|  | Alcohol Abuse | 0.44 | 0.04 | -10.40 | | 0.000 | | 0.36 | | 0.53 | |  |
|  | Drug Abuse | 0.51 | 0.04 | -11.36 | | 0.000 | | 0.42 | | 0.60 | |  |
|  | Depression | -0.08 | 0.03 | 2.65 | | 0.008 | | -0.15 | | -0.02 | |  |
|  | Anxiety disorder | 0.04 | 0.04 | -0.84 | | 0.399 | | -0.05 | | 0.12 | |  |
|  | Somatoforme Disorder | 0.04 | 0.04 | -0.97 | | 0.332 | | -0.04 | | 0.12 | |  |

*Notes:* SE standard error, CI Confidence interval, all numbers were rounded to 2 decimal places (p values to 3 decimal places)

| **Table 4c coefficients of the GLMM for binary data (**$\boldsymbol{FC}_{\boldsymbol{p}}$ **weights)** | | | | | | | | |  |
| --- | --- | --- | --- | --- | --- | --- | --- | --- | --- |
| **Category** | **Variable** |  | **Estimate** | **SE** | **Statistic** | **p** | **CI lower** | **CI upper** | |
| Connection Type | GP – GP | Low | 0.12 | 0.04 | -2.75 | 0.006 | 0.03 | 0.20 | |
|  |  | Middle | 0.05 | 0.06 | -0.89 | 0.375 | -0.06 | 0.16 | |
|  |  | High | -0.02 | 0.06 | 0.34 | 0.731 | -0.13 | 0.09 | |
|  | NP – GP | Low | -0.22 | 0.06 | 3.97 | 0.000 | -0.33 | -0.11 | |
|  |  | Middle | -0.33 | 0.06 | 5.28 | 0.000 | -0.46 | -0.21 | |
|  |  | High | -0.48 | 0.06 | 7.31 | 0.000 | -0.60 | -0.35 | |
|  | NP – NP | Low | 0.22 | 0.08 | -2.74 | 0.006 | 0.06 | 0.37 | |
|  |  | Middle | 0.26 | 0.20 | -1.30 | 0.192 | -0.13 | 0.66 | |
|  |  | High | 0.01 | 0.22 | -0.04 | 0.968 | -0.42 | 0.44 | |
|  | N – GP | Low | -0.22 | 0.09 | 2.43 | 0.015 | -0.40 | -0.04 | |
|  |  | Middle | -0.31 | 0.11 | 2.73 | 0.006 | -0.53 | -0.09 | |
|  |  | High | -0.28 | 0.11 | 2.45 | 0.015 | -0.49 | -0.06 | |
|  | N – NP | Low | -0.18 | 0.21 | 0.89 | 0.374 | -0.59 | 0.22 | |
|  |  | Middle | 0.15 | 0.23 | -0.66 | 0.508 | -0.30 | 0.61 | |
|  |  | High | -0.68 | 0.34 | 2.04 | 0.042 | -1.34 | -0.03 | |
|  | N – N | Low | 0.06 | 0.14 | -0.42 | 0.677 | -0.21 | 0.33 | |
|  |  | Middle | -0.06 | 0.40 | 0.16 | 0.873 | -0.84 | 0.71 | |
|  |  | High | 0.14 | 0.35 | -0.41 | 0.684 | -0.54 | 0.82 | |
|  | Psy – GP | Low | -0.29 | 0.06 | 5.07 | 0.000 | -0.41 | -0.18 | |
|  |  | Middle | -0.37 | 0.07 | 5.06 | 0.000 | -0.51 | -0.22 | |
|  |  | High | -0.42 | 0.07 | 5.68 | 0.000 | -0.56 | -0.27 | |
|  | Psy – NP | Low | 0.47 | 0.13 | -3.62 | 0.000 | 0.21 | 0.72 | |
|  |  | Middle | 0.12 | 0.18 | -0.66 | 0.509 | -0.24 | 0.48 | |
|  |  | High | -0.19 | 0.21 | 0.92 | 0.357 | -0.59 | 0.21 | |
|  | Psy – N | Low | -0.12 | 0.24 | 0.52 | 0.604 | -0.59 | 0.34 | |
|  |  | Middle | -0.15 | 0.33 | 0.46 | 0.647 | -0.79 | 0.49 | |
|  |  | High | -0.35 | 0.35 | 1.01 | 0.312 | -1.03 | 0.33 | |
|  | Psy – Psy | Low | 0.18 | 0.09 | -1.94 | 0.052 | 0.00 | 0.36 | |
|  |  | Middle | 0.28 | 0.24 | -1.15 | 0.248 | -0.19 | 0.75 | |
|  |  | High | -0.10 | 0.26 | 0.39 | 0.699 | -0.62 | 0.42 | |
|  | PT – GP | Low | -0.12 | 0.12 | 0.97 | 0.330 | -0.36 | 0.12 | |
|  |  | Middle | -0.26 | 0.38 | 0.69 | 0.488 | -1.00 | 0.48 | |
|  |  | High | 0.13 | 0.31 | -0.43 | 0.669 | -0.47 | 0.73 | |
|  | PT – NP | Low | -0.04 | 0.20 | 0.20 | 0.842 | -0.43 | 0.35 | |
|  |  | Middle | 0.43 | 0.37 | -1.17 | 0.241 | -0.29 | 1.16 | |
|  |  | High | -0.11 | 0.44 | 0.24 | 0.808 | -0.97 | 0.76 | |
|  | PT – Psy | Low | 0.26 | 0.19 | -1.40 | 0.160 | -0.10 | 0.63 | |
|  |  | Middle | -0.58 | 0.48 | 1.21 | 0.226 | -1.51 | 0.36 | |
|  |  | High | -0.48 | 0.48 | 0.99 | 0.320 | -1.42 | 0.46 | |
|  |  | Low | -0.13 | 0.31 | 0.40 | 0.688 | -0.74 | 0.49 | |
|  | PT – N | Middle | 0.22 | 0.58 | -0.37 | 0.712 | -0.93 | 1.36 | |
|  |  | High | -0.97 | 1.04 | 0.93 | 0.350 | -3.01 | 1.07 | |

*Notes:* GP general practitioner, NP neurologist/psychiatrist, Psy psychiatrist, PT psychotherapist, SE standard error, CI Confidence interval, all numbers were rounded to 2 decimal places (p values to 3 decimal places), the categories low, middle, high indicate that the corresponding connection fell into the first, second or third tertile of the connection type specific distribution of the number of shared patients

## **Results of the GLMM to predict hospital admission in the second year of the follow-up:**

| **Table 5a: coefficients of the GLMM for binary data (Elixhauser subscales)** | | | | |
| --- | --- | --- | --- | --- |
|  | **Variable** | **Model 1 (baseline)** | **Model 2**  **(incl. CD)** | **Model 3**  **(incl. FCD)** |
| Elixhauser-subscales | Congestive heart failure | 0.14* | 0.15* | 0.16* |
|  | Cardiac arrythmias | 0.14* | 0.15** | 0.14* |
|  | Valvular disease | -0.29* | -0.31** | -0.36** |
|  | Pulmonary circulation disorders | 0.28 | 0.28 | 0.29 |
|  | Vascular disorders | 0.28*** | 0.28*** | 0.32*** |
|  | Diabetes (complicated) | 0.03 | 0.04 | 0.05 |
|  | Diabetes (uncomplicated) | -0.15 | -0.15 | -0.19* |
|  | Paralysis | -0.02 | -0.02 | -0.04 |
|  | Neurodegenerative disorders | 0.08 | 0.10 | 0.09 |
|  | Chronic pulmonary disease | 0.10** | 0.12** | 0.11* |
|  | Hypertension (complicated) | 0.13* | 0.13 | 0.14* |
|  | Hypertension (uncomplicated) | -0.02 | 0.00 | -0.01 |
|  | Hypothyroidism | -0.02 | -0.02 | -0.01 |
|  | Renal failure | -0.14 | -0.14 | -0.19* |
|  | Chronic pulmonary disease | -0.04 | -0.06 | -0.04 |
|  | Solid tumour without metastasis | -0.03 | -0.04 | -0.03 |
|  | Rheumatoid arthritis/CVD | -0.06 | -0.06 | -0.07 |
|  | Coagulopathy | -0.05 | -0.04 | -0.05 |
|  | Obesity | 0.07 | 0.08 | 0.08 |
|  | Weight loss | -0.02 | -0.02 | -0.02 |
|  | Fluid and electrolyte diseases | 0.43*** | 0.46*** | 0.45*** |
|  | Blood loss anemia | 0.23 | 0.25 | 0.26 |
|  | Deficiency anemia | 0.09 | 0.09 | 0.10 |
|  | Alcohol Abuse | 0.42*** | 0.45*** | 0.45*** |
|  | Drug Abuse | 0.50*** | 0.55*** | 0.52*** |
|  | Depression | -0.11** | -0.11** | -0.10** |
|  | Anxiety disorder | 0.05 | 0.05 | 0.06 |
|  | Somatoform disorder | 0.03 | 0.04 | 0.03 |

*Notes:* all numbers were rounded to 2 decimal places, *p<.05, **p<.01, ***p<.001

| **Table 5b: coefficients of the GLMM for binary data (all other predictors)** | | | | |
| --- | --- | --- | --- | --- |
|  | **Variable** | **Model 1 (baseline)** | **Model 2**  **(incl. CD)** | **Model 3**  **(incl. FCD)** |
| Other control variables | Hospitalization | 1.67*** | 1.64*** | 1.62*** |
|  | Antipsychotics | 0.35*** | 0.40*** | 0.39*** |
|  | Care level 0 | 0.37*** | 0.38*** | 0.40*** |
|  | Care level 1 | 0.17** | 0.18** | 0.19** |
|  | Care level 2 | 0.15 | 0.14 | 0.18 |
|  | Care level 3 | -0.11 | -0.12 | -0.12 |
|  | Care level 4 | 0.50 | 0.48 | 0.47 |
|  | Nursing home | 0.18 | 0.18 | 0.22* |
|  | Age | -0.02*** | -0.02*** | -0.02*** |
|  | Sex | 0.03 | 0.03 | 0.04 |
|  | residency in a big city | -0.06 | -0.05 | -0.05 |
|  | residency in an urban area | -0.04 | -0.03 | -0.04 |
|  | Neurologist contacts | -0.02** | -0.01** | -0.01* |
|  | Psychiatrists contacts | 0.00 | 0.00 | 0.00 |
|  | Psychotherapist contacts | 0.00 | 0.00 | 0.00 |
| Coordination metrics | Care density |  | 0.00 |  |
|  | Care density (missing) |  | 0.10** |  |
|  | Fragmented Care Density |  |  | 0.61*** |
| Other model parameters | Intercept | -2.85*** | -3.11*** | -1.31*** |
|  | Patient-level variance | 0.44*** | 0.42*** | 0.42*** |
| Global Fit Statistics | -2 Log Likelihood | 34590.69 | 34580.26 | 34537.39 |
|  | AIC | 34680.69 | 34674.26 | 34629.39 |
|  | BIC | 35038.57 | 35048.05 | 34995.23 |

*Notes:* all numbers were rounded to 2 decimal places, *p<.05, **p<.01, ***p<.001, Care density (missing) is a dummy indicator that encodes whether care density can be calculated (i.e. whether the patient was treated by at least two outpatient providers).

1. Frey, S.J.E.P., *The economic burden of schizophrenia in Germany: a population-based retrospective cohort study using genetic matching.* 2014. **29**(8): p. 479-489.

2. Häcker, J. and T.J.H.e. Hackmann, *Los (T) In Long‐Term Care: Empirical Evidence From German Data 2000–2009.* 2012. **21**(12): p. 1427-1443.

3. Lorenz, M.O.J.P.o.t.A.s.a., *Methods of measuring the concentration of wealth.* 1905. **9**(70): p. 209-219.
